# Predictors of Mental Health Literacy in a Sample of Health Care Major Students: Pilot Evaluation Study

**DOI:** 10.2196/43770

**Published:** 2024-02-08

**Authors:** Pia Tohme, Nour Abi Fadel, Nour Yaktine, Rudy Abi-Habib

**Affiliations:** 1 Department of Social and Education Sciences Lebanese American University Beirut Lebanon; 2 Department of Psychology Saint Joseph University of Beirut Beirut Lebanon; 3 American University of Beirut Beirut Lebanon

**Keywords:** awareness, COVID-19, digital health literacy, digital health, disorder, empathy, health literacy, literacy, mental health literacy, mental health, mentalizing, questionnaire, students

## Abstract

**Background:**

The numerous mental health awareness campaigns during the COVID-19 pandemic have shifted our understanding and perception of mental health.

**Objective:**

The purpose of this study is to evaluate predictors of mental health literacy (MHL), that is, one’s knowledge and beliefs about mental disorders. We evaluate whether digital health literacy, empathy, and mentalizing contribute to MHL.

**Methods:**

Our sample consisted of 89 health care major students, aged between 17 and 32 years, studying at a university in Lebanon. The Mental Health Literacy Scale for Healthcare Students (MHLS-HS), the eHealth Literacy Questionnaire (eHLQ), the Basic Empathy Scale (BES), and the Reflective Functioning Questionnaire-8 (RFQ-8) were used.

**Results:**

Multiple regression analyses revealed that the Engagement in Own Health subscale of digital health literacy constituted a predictor of MHL. While empathy and mentalizing did not directly predict MHL, they were found to predict components of MHL.

**Conclusions:**

This is the first study to evaluate digital health literacy, empathy, and mentalizing as predictors of MHL in Lebanon, a country where mental health is still considered taboo. Moreover, this pilot study is the first to provide some support for the predictive role of some digital health literacy subscales on MHL in light of the rise of the digital era following the COVID-19 pandemic.

## Introduction

### Overview

Despite the negative impact of the COVID-19 pandemic on both physical and mental health, the global health crisis has reshaped our perception and understanding of mental health, reducing mental health stigma and normalizing mental health-related discussions [1]. In a way, the COVID-19 era has promoted mental health literacy (MHL), a concept defined as our “knowledge and beliefs about mental disorders that aid their recognition, management, or prevention” [2]. MHL has been found to enhance self-efficacy for help-seeking, diminish stigma surrounding mental disorders, and, most importantly, increase the ability to maintain psychological well-being [3]. Therefore, it could be argued that promoting MHL could play a central role in preventing and treating mental disorders. Most studies evaluating MHL focus on the general public’s understanding of mental health rather than factors that can contribute to MHL. Addressing this gap, this study aims to explore predictors of MHL in a sample of health care major students. More specifically, we evaluate whether the availability of practical tools such as digital health technologies or psychological characteristics such as empathy or mentalizing can contribute to MHL in health care major students.

### The Role of Digital Health in Promoting MHL

In today’s digital age, health care providers have been increasingly making use of technology in their professional practice [4]. The use of digital health has increased at an even higher rate during the COVID-19 pandemic, as it has become a necessity at the individual, institutional, and social levels [5,6]. Beyond being a highly effective solution to working through health problems during the pandemic, digital health—in other words, accessing health-related information on the web—has the potential to shift the health care paradigm from mere treatment to prevention [7,8]. In Lebanon, Tohme et al [9] found that mental health professionals already had experience delivering web-based consultations before the COVID-19 pandemic, and, despite preferring face-to-face sessions, mental health practitioners reported numerous benefits of using digital health tools.

Initially adopted as a short-term solution to overcome obstacles imposed during the COVID-19 pandemic (eg, lockdown), the use of digital technologies for health has enhanced digital health literacy [10], meaning the ability to seek, find, understand, and assess health-related information from electronic sources in order to address and solve a health problem [11]. Digital health literacy allows individuals to communicate health information and make informed decisions that promote well-being. In a systematic review evaluating the association between health literacy, digital health literacy, and physical health outcomes [12], digital health literacy was found to be associated with perceived and reported better communication with health care providers, health-promoting behaviors, and self-management of health needs [13,14]. As for mental health, Lincoln et al [15] found that low health literacy was associated with negative mental health outcomes such as depressive symptoms. Whether these results apply to digital health literacy is still unknown. Research has yet to establish an association between digital health literacy and mental health outcomes, an association that could be mediated by MHL.

As an extension of health literacy, MHL is a relatively new construct, focusing specifically on accessing web-based information about mental health, namely emotional and psychological difficulties [2]. Hence, most studies on digital health literacy focus on its association with physical health outcomes only. To our knowledge, no study has yet evaluated whether this association applies to mental health. It can be argued, however, that digital health literacy can be a crucial component in promoting MHL. Understanding the potential benefits of digital health literacy in promoting MHL would be useful for policymakers, as it would allow them to devise policies and interventions focusing on promoting the use of digital health in the health care sector. This would equip health care providers with the tools needed to work in a digitized health sector [16,17], contributing to MHL in the general public and potentially leading to better psychological outcomes.

### Empathy and Mentalizing as Predictors of MHL

Another line of research focused on pinpointing the psychological factors that could predict MHL, such as empathy. Empathy, or putting oneself in others’ shoes, is understood as one’s ability to understand what the other is feeling and to match that emotion [18]. As a concept, empathy entails both cognitive and affective elements that lead to a sense of emotional understanding [19]. The emotional component of empathy relates to one’s emotional response to the other’s experience, while cognitive empathy involves imagining the other person’s mental state, feelings, and perspective. Both components have been shown to generate a more positive attitude toward mental disorders [20] and are important components of MHL [21]. Indeed, evaluating the association between empathy and MHL, Furnham and Sjokvist [22] established a positive correlation between the 2 constructs, showing that individuals who experience empathy are more likely to be knowledgeable about mental disorders, as they tend to be more interested in reading and learning about mental disorders. Mendenhall and Frauenholtz [23] have also shown that having children diagnosed with mood disorders such as bipolar disorder could promote MHL. More recently, Piper et al [24] confirmed this association by showing that in older people, being in close proximity to someone with a mental disorder predicted MHL. These findings show that familiarity with mental disorders and the ability to recognize mental disorder symptoms could be influenced by one’s personal experience with one’s children and loved ones, their curiosity to know more about mental disorders, and possibly other factors, such as being emotionally present for others. It can be argued that, in turn, this familiarity could decrease stigma toward mental disorders and increase the level of knowledge of symptoms and their appropriate treatments. Hence, exposure to mental disorders (eg, knowing someone with a mental disorder) could relate to greater knowledge and appreciation of mental illness [21], hence being significantly correlated to MHL.

Although empathy and mentalizing are similar constructs, mentalizing refers to one’s ability to envision one’s own mental states as well as others’. These include feelings, thoughts, intentions, and beliefs focusing on a more interpersonal level, thinking about how others’ feelings are affecting us, and how, in turn, this can modify our response to them [25]. Mentalizing does not necessarily entail empathy, as research shows that people diagnosed with psychosis are often unable to empathize with others but are capable of mentalizing [26]. Moreover, although empathy contains a cognitive component, cognitive empathy relates to one’s ability to attribute emotions rather than cognitions, such as in the case of mentalizing. Although the 2 constructs are dissociable in nature, both have common underlying features. As such, it could be argued that mentalizing, just like empathy, could also predict MHL. Indeed, the ability to understand others’ minds and mental states can be central to promoting MHL, since understanding others’ mental states could also mean understanding their mental health status and experiences. Given the fact that displaying empathy has been shown to be associated with increased MHL [21] and that empathy and mentalization are similar constructs in nature, an association between mentalization and MHL has thus been hypothesized. However, this association has yet to be established in the literature.

MHL is a relatively new concept, hence the gap in the literature as to what contributes to its development. Indeed, the few studies evaluating the predictors of MHL have mainly focused on demographic factors such as age, gender, and level of education [23]. For instance, in Lebanon, the only study evaluating predictors of MHL in a sample of university students revealed that education in psychology was a strong predictor of MHL [27]. However, given the importance of digital health literacy in promoting well-being, this pilot study aims to understand whether digital health literacy contributes to MHL in health care major students. Moreover, we aim to explore psychological factors such as empathy and mentalizing as predictors of MHL. Evaluating these predictors in a population of health care major students would help set the stepping stone for future research, aiming to understand whether interventions and trainings are needed to promote the use of digital health in the health care system. Finally, our results could provide initial support in identifying psychological features that promote MHL in order for health care professionals to not only self-cultivate these features but also target these elements when working with patients in order to increase MHL and promote psychological well-being.

## Methods

### Participants

In this study, purposive sampling was used, and the study sample consisted of 89 university students. Participants consisted of both undergraduate and graduate health care major students (ie, psychology, premed tracks, nutrition, pharmacy, and medicine), recruited from the Lebanese American University, a private university in Beirut, Lebanon. Students were aged between 17 and 32 (mean 19.64, SD 2.01) years, and most of the sample (66/89, 75%) identified as women. Inclusion criteria included being a Lebanese university student, being aged 18 years or older, and being fluent in English.

### Measures

In order to evaluate whether digital health literacy, empathy, and mentalizing predicted our outcome variable (ie, MHL), 4 questionnaires were used. Demographic variables that were collected included age, gender, and academic major.

The Mental Health Literacy Scale for Healthcare Students (MHLS-HS) [3] is a self-report questionnaire used to measure MHL in health care major students. Rated on a 5-point Likert scale (1=strongly disagree and 5=strongly agree), the MHLS-HS comprises 26 items and the following 5 subscales: maintenance of positive mental health (10 items), recognition of mental illness (4 items), attitude to mental illness stigma (6 items), help-seeking efficacy (3 items), and help-seeking attitude (3 items). The MHLS-HS is scored by summing the item scores, with higher scores indicating a better MHL. The MHLS-HS has shown good internal consistency, with α values ranging between .70 and .90 across subscales [3].

The eHealth Literacy Questionnaire (eHLQ) [27] is a self-report questionnaire that measures digital health literacy. The 35-item scale is rated on a 4-point Likert scale (1=strongly disagree and 4=strongly agree) and consists of seven subscales measuring the following components of digital health literacy: (1) using technology to process health information, (2) engagement in own health, (3) ability to actively engage with digital services, (4) the ability to feel safe and in control, (5) motivation to engage with digital services, (6) access to digital services that work, and (7) digital services that suit individual needs. Each subscale contains 5 items, except for subscale 6 containing 6 items and subscale 7 containing 4 items. The average score is calculated for each subscale, with higher scores indicating better digital health literacy. The eHLQ has been found to have good psychometric properties with composite reliability above 0.7 for all 7 subscales [27].

The Basic Empathy Scale (BES) [28] is a 20-item self-report scale that is used to measure empathy in its cognitive and affective elements. The first factor relates to cognitive empathy and is comprised of 9 items, while the second factor relates to affective empathy and is comprised of 11 items. Items are rated on a 5-point Likert scale (1=strongly disagree and 5=strongly agree). Scores are calculated by computing the average for each subscale, with higher scores indicating higher self-reported empathy. Overall empathy is calculated by summing the averages of the 2 subscales. The BES has demonstrated good internal consistency, with α=.79 for the cognitive empathy subscale and α=.85 for the affective empathy subscale [28].

The Reflective Functioning Questionnaire-8 (RFQ-8) [29] originally consisted of a 54-item self-report scale measuring mentalizing capacities. It is rated on a 7-point Likert scale (1=strongly disagree and 7=strongly agree) and is comprised of 2 subscales: uncertainty about mental states (RFQu) and certainty about mental states (RFQc). High scores on RFQc and low scores on RFQu reflect genuine mentalizing, while low scores on RFQc reflect hypermentalizing and high scores on RFQu reflect hypomentalizing, both indicating failure to mentalize. The RFQ-54 has demonstrated good internal consistency, with α=.67 for RFQc and α=.63 for RFQu. A shorter version of the RFQ-54, consisting of 8 items, was created by Fonagy et al [29] for research purposes and was used in this study.

### Procedure

Data collection took place between October 2020 and December 2020, at a time when Lebanon was under lockdown due to the COVID-19 pandemic. Hence, data collection took place on the web, using Google Forms. Participation took between 15 and 20 minutes to complete.

### Data Analysis

The aim of this exploratory pilot study was to investigate predictors of MHL. For this purpose, we ran a hierarchical multiple regression with MHL as the dependent variable and digital health literacy (eHLQ, Model 1), empathy (eHLQ and BES, Model 2), and mentalizing (eHLQ, BES, and RFQ, Model 3) as the independent variables. SPSS (SPSS Inc) was used for all analyses.

Moreover, since the literature shows that MHL entails recognition of mental illness, leading to lower stigma toward mental health, as well as help-seeking behaviors [2,3], we ran 3 additional hierarchical multiple regressions with the “Recognition of Mental Illness,” “Attitude Toward Mental Illness Stigma,” and “Help-Seeking Attitude” subscales as the dependent variables, and digital health literacy (eHLQ, Model 1), empathy (eHLQ and BES, Model 2), and mentalizing (eHLQ, BES, and RFQ, Model 3) as the independent variables.

### Ethical Considerations

This study received ethical approval from the university institutional review board (LAU.SAS.PT5.27/Oct/2020). The survey was circulated on social media, including an information sheet. Participants interested in taking part e-signed the consent form before accessing the questionnaires. All data were anonymous, with no identifiers linking responses to the participant’s identity. Participants were informed that participation is voluntary and that they could drop out at any time. There was no compensation for participation.

## Results

The hierarchical regression analysis predicting MHL revealed that in the first model, “Engagement in Own Health” was a significant predictor of MHL, with *F*_7,81_=2.19; *P*=.04 and accounted for 16% of the variation in MHL. Introducing empathy (Model 2) explained an additional 4% of variation, though the model was not statistically significant (*F*_2,79_=1.75; *P*=.18). Finally, introducing mentalizing (Model 3) did not explain any additional variation in the model, *F*_2,77_=0.23; *P*=.80 (Table 1).

The hierarchical regression looking for predictors of the “Recognition of Mental Illness” subscale of MHL revealed that the first model, digital health literacy, was not significant (*F*_7,81_=9.60; *P*=.47). Introducing the 2 empathy subscales explained an additional 8% of variation, and this change in *R*² was significant (*F*_2,79_=3.48; *P*=.04), with affective empathy found to be a significant predictor. Adding the 2 mentalizing subscales to the regression model explained an additional 7% of variation, and this change in *R*² was significant (*F*_2,77_=3.58; *P*=.03), with affective empathy and RFQc found to be significant predictors. Together, all factors explained 22% of the variation in the “Recognition of Mental Illness” subscale (Table 2).

The hierarchical regression looking for predictors of the “Attitude to Mental Illness Stigma” subscale revealed that the first model, including the digital health literacy subscales, was not significant (*F*_7,81_=1.83; *P*=.09). Introducing the 2 empathy subscales also led to a nonsignificant model (Model 2) with *F*_9,79_=2.09; *P*=.05. When all factors were included (Model 3), they were found to significantly predict 27% of the variation in the “Attitude to Mental Illness Stigma” subscale (*F*_11,77_=2.54; *P*=.009), with the “Being Motivated to Engage with Digital Services” subscale of digital health literacy and RFQu found to be significant predictors (Table 3).

The hierarchical regression looking for predictors of the “Help-Seeking Attitude” subscale of MHL revealed that in the first model, the “Ability to Process Information,” “Engagement in Own Health,” “Feeling Safe and in Control,” and “Being Motivated to Engage with Digital Services” subscales of digital health literacy were significant predictors in the regression model (*F*_7,81_=4.28; *P*<.001) and accounted for 27% of the variation in the model. Introducing the 2 empathy subscales (Model 2) and the 2 mentalizing subscales (Model 3) did not lead to significant changes in *R*² with *F*_2,79_=0.1; *P*=.89 and *F*_2,77_=1.12; *P*=.31, respectively (Table 4).

**Table 1:** Summary of hierarchical regression analysis for variables predicting mental health literacy (MHL) total score.

Variable		B	SE	β	*t*	*R*		*R* ^2^		Δ*R*^2^		*P* value
**Model 1**							0.39		0.16		0.16	.04
	Ability to process information	–3.72	3.31	–.20	–1.12							.26
	Engagement in own Health	7.56	2.82	.36	2.68							.009
	Ability to actively engage with digital services	.92	2.79	.05	0.33							.74
	Feel safe and in control	2.67	2.09	.15	1.28							.20
	Motivated to engage with digital services	1.39	3.06	.08	0.46							.65
	Access to digital services that work	–2.99	2.69	–.16	–1.11							.27
	Digital services that suit individual needs	2.08	3.09	.12	0.68							.50
**Model 2**							0.44		0.19		0.04	.18
	Ability to process information	–3.01	3.31	–.16	–0.91							.36
	Engagement in own Health	6.64	2.84	.31	2.33							.02
	Ability to actively engage with digital services	–0.03	2.82	–.00	–0.01							.99
	Feel safe and in control	2.47	2.08	.14	1.19							.24
	Motivated to engage with digital services	1.31	3.04	.08	0.43							.67
	Access to digital services that work	–1.26	2.83	–.07	–0.45							.65
	Digital services that suit individual needs	0.31	3.23	.02	0.09							.92
	Affective empathy	–3.23	1.73	–.24	–1.87							.06
	Cognitive empathy	1.58	1.53	.13	1.03							.30
**Model 3**							0.45		0.20		0.00	.80
	Ability to process information	–2.55	3.42	–.14	–0.75							.46
	Engagement in own Health	6.86	2.89	.32	2.37							.02
	Ability to actively engage with digital services	0.01	2.91	.00	0.00							.99
	Feel safe and in control	2.46	2.15	.14	1.14							.25
	Motivated to engage with digital services	1.37	3.07	.08	0.45							.66
	Access to digital services that work	–1.19	2.94	–.06	–0.41							.68
	Digital services that suit individual needs	–0.21	3.36	–.01	–0.06							.95
	Affective empathy	–2.73	1.90	–.20	–1.43							.15
	Cognitive empathy	1.05	1.78	.08	0.59							.56
	Reflective functioning: certainty subscale	0.35	2.82	.02	0.12							.90
	Reflective functioning: uncertainty subscale	–1.31	2.20	–.08	–0.59							.55

**Table 2:** Summary of hierarchical regression analysis for variables predicting the Recognition of Mental Illness Subscale Score.

Variable		B	SE	β	*t*	*R*		*R* ^2^		Δ*R*^2^		*P* value	
**Model 1**							0.28		0.08		0.08		.47
	Ability to process information	–0.11	0.21	–.09	–0.53							.59	
	Engagement in own Health	–0.18	0.18	–.14	–1.02							.31	
	Ability to actively engage with digital services	0.18	0.18	.16	1.03							.30	
	Feel safe and in control	0.04	0.13	.03	0.27							.79	
	Motivated to engage with digital services	0.06	0.19	.06	0.31							.76	
	Access to digital services that work	–0.32	0.17	–.29	–1.89							.06	
	Digital services that suit individual needs	0.13	0.19	.12	0.65							.52	
**Model 2**							0.39		0.15		0.08		.04
	Ability to process information	–0.05	0.21	–.05	–0.26							.79	
	Engagement in own Health	–0.27	0.18	–.21	–1.50							.14	
	Ability to actively engage with digital services	0.10	0.18	.09	0.59							.56	
	Feel safe and in control	0.02	0.13	.01	0.12							.91	
	Motivated to engage with digital services	0.06	0.19	.06	0.29							.77	
	Access to digital services that work	–0.18	0.18	–.16	–1.00							.32	
	Digital services that suit individual needs	–0.02	0.20	–.02	–0.09							.92	
	Affective empathy	–0.28	0.11	–.35	–2.63							.01	
	Cognitive empathy	0.11	0.09	.15	1.19							.23	
**Model 3**							0.47		0.22		0.07		.03
	Ability to process information	–0.04	0.20	–.04	–0.22							.83	
	Engagement in own Health	–0.31	0.17	–.24	–1.78							.08	
	Ability to actively engage with digital services	0.01	0.17	.01	0.04							.97	
	Feel safe and in control	–0.05	0.13	–.05	–0.40							.69	
	Motivated to engage with digital services	0.05	0.18	.05	0.27							.79	
	Access to digital services that work	–0.08	0.18	–.07	–0.44							.66	
	Digital services that suit individual needs	–0.02	0.20	–.02	–0.11							.91	
	Affective empathy	–0.32	0.11	–.39	–2.79							.007	
	Cognitive empathy	0.09	0.11	.12	0.86							.39	
	Reflective functioning: certainty subscale	0.39	0.17	.31	2.38							.02	
	Reflective functioning: uncertainty subscale	0.24	0.13	.24	1.85							.07	

**Table 3:** Summary of hierarchical regression analysis for variables predicting the Attitude to Mental Illness Stigma Subscale Score.

Variable		B	SE	β	*t*	*R*		*R* ^2^		Δ*R*^2^		*P* value	
**Model 1**							0.37		0.14		0.14		.09
	Ability to process information	–0.00	0.27	.00	–0.00							.99	
	Engagement in own Health	0.12	0.23	.07	0.53							.60	
	Ability to actively engage with digital services	0.19	0.23	.13	0.86							.39	
	Feel safe and in control	–0.18	0.17	–.12	–1.01							.32	
	Motivated to engage with digital services	–0.65	0.25	–.48	–2.57							.01	
	Access to digital services that work	–0.15	0.22	–.09	–0.68							.49	
	Digital services that suit individual needs	0.35	0.26	.24	1.36							.18	
**Model 2**							0.44		0.19		0.06		0.5
	Ability to process information	–0.01	0.27	–.01	–0.05							.96	
	Engagement in own Health	0.19	0.23	.11	0.85							.40	
	Ability to actively engage with digital services	0.23	0.23	.15	0.99							.33	
	Feel safe and in control	–0.13	0.17	–.09	–0.77							.44	
	Motivated to engage with digital services	–0.68	0.25	–.49	–2.73							.008	
	Access to digital services that work	–0.24	0.23	–.16	–1.03							.31	
	Digital services that suit individual needs	0.39	0.27	.27	1.47							.15	
	Affective empathy	0.20	0.14	.18	1.43							.16	
	Cognitive empathy	0.11	0.13	.10	0.84							.40	
**Model 3**							0.52		0.27		0.07		.009
	Ability to process information	0.12	0.27	.08	0.45							.66	
	Engagement in own Health	0.28	0.23	.16	1.22							.23	
	Ability to actively engage with digital services	0.27	0.23	.18	1.19							.24	
	Feel safe and in control	–0.11	0.17	–.08	–0.67							.51	
	Motivated to engage with digital services	–0.66	0.24	–.48	–2.74							.008	
	Access to digital services that work	–0.25	0.23	–.16	–1.09							.28	
	Digital services that suit individual needs	0.24	0.26	.17	0.90							.37	
	Affective empathy	0.36	0.15	.33	2.43							.02	
	Cognitive empathy	–0.04	0.14	–.04	–0.32							.75	
	Reflective functioning: certainty subscale	–0.03	0.22	–.02	–0.15							.88	
	Reflective functioning: uncertainty subscale	–0.47	0.17	–.34	–2.71							.008	

**Table 4:** Summary of hierarchical regression analysis for variables predicting the Help-Seeking Attitude Subscale Score.

Variable			B		SE B		β		*t*		*R*			*R* ^2^			Δ*R*^2^			*P* value	
**Model 1**												0.52			0.27			0.27			<.001
	Ability to process information	–0.63		0.29		–.35		–2.13											.04		
	Engagement in own Health	0.53		0.25		.26		2.09											.04		
	Ability to actively engage with digital services	–0.26		0.25		–.14		–1.05											.29		
	Feel safe and in control	0.59		0.19		.36		3.21											.002		
	Motivated to engage with digital services	0.66		0.27		.41		2.41											.02		
	Access to digital services that work	0.39		0.24		.22		1.61											.11		
	Digital services that suit individual needs	–0.30		0.27		–.18		–1.09											.27		
**Model 2**												0.52			0.27			0.00			.89
	Ability to process information	–0.63		0.29		–.36		–2.10											.04		
	Engagement in own Health	0.54		0.26		.27		2.11											.04		
	Ability to actively engage with digital services	–0.25		0.26		–.14		–0.99											.32		
	Feel safe and in control	0.61		0.19		.36		3.21											.002		
	Motivated to engage with digital services	0.65		0.28		.41		2.36											.02		
	Access to digital services that work	0.37		0.26		.21		1.43											.15		
	Digital services that suit individual needs	–0.29		0.29		–.18		–0.99											.32		
	Affective empathy	0.05		0.16		.04		0.29											.77		
	Cognitive empathy	0.02		0.14		.02		0.17											.86		
**Model 3**												0.54			0.29			0.02			.31
	Ability to process information	–0.72		0.31		–.41		–2.35											.02		
	Engagement in own Health	0.53		0.26		.26		2.04											.04		
	Ability to actively engage with digital services	–0.19		0.26		–.11		–0.76											.45		
	Feel safe and in control	0.65		0.19		.39		3.38											.001		
	Motivated to engage with digital services	0.64		0.28		.40		2.34											.02		
	Access to digital services that work	0.29		0.26		.16		1.10											.27		
	Digital services that suit individual needs	–0.19		0.30		–.12		–0.64											.52		
	Affective empathy	–0.03		0.17		–.02		–0.15											.88		
	Cognitive empathy	0.14		0.16		.12		0.87											.39		
	Reflective functioning: certainty subscale	–0.33		0.25		–.16		–1.29											.20		
	Reflective functioning: uncertainty subscale	0.08		0.19		.05		0.44											.66		

## Discussion

With the global health crisis of the COVID-19 pandemic and the numerous lockdowns across the globe, health care providers have recently switched to web-based health care. Moreover, with the increase in mental health awareness campaigns, the COVID-19 era has drastically shifted our perception of mental health, reducing the stigma surrounding mental disorders. In this digital age of mental health awareness, this pilot study aimed to explore predictors of MHL in health care major students, specifically evaluating whether digital health literacy contributes to MHL. Moreover, we explored psychological factors such as empathy and mentalizing in an attempt to pinpoint predictors of MHL.

Our results suggest that “Engagement in Own Health,” a digital health literacy component, is a predictor of overall MHL. This confirms previous findings showing that health engagement improves patient activation, meaning patients’ ability to manage their health [30]. This ability goes hand in hand with MHL, as it entails recognizing, managing, and seeking treatment for mental disorders [2]. Moreover, the “Ability to Process Information,” “Engagement in Own Health,” “Feeling Safe and in Control,” and “Being Motivated to Engage with Digital Services” subscales of digital health literacy were found to predict MHL components. This partially confirms our hypothesis on the role of digital health literacy in promoting MHL, indicating that some constructs of digital health play a vital role in promoting MHL. In that sense, digital health could be hypothesized to play a role in decreasing stigma surrounding mental disorders, allowing individuals to become more open to seeking help. This is especially important in a country such as Lebanon, where mental health remains taboo, thus preventing individuals from seeking help in an attempt to avoid criticism [31]. Our results hint at the importance of digital health practices and services in the health care system and call for future research to further explore whether specialized digital health trainings for health care major students as well as the general public could increase MHL. This is of special importance given the cost-effectiveness of digital services, making them more easily implemented, particularly given the current socioeconomic crisis in Lebanon.

While empathy has been shown to contribute to MHL [21,22], our results did not fully support this hypothesis. It is worth noting, however, that both empathy and mentalizing predicted components of MHL, namely “Recognition of Mental Illness” and “Attitude to Mental Illness Stigma.” This partially confirms findings on the role of empathy in promoting MHL [22-24]. Moreover, these findings support our hypothesis on the role of mentalizing in promoting MHL, a relationship that, to our knowledge, was never explored in the literature. Indeed, mentalizing refers to one’s capacity to think in terms of mental states underlying behaviors [25]; it can therefore be argued that this capacity facilitates people’s awareness of difficulties with emotion regulation, thus pinpointing signs of distress or mental health problems. Given that mentalizing was found to play a protective factor against stigma as it promotes thinking about the potential negative effects of being subjected to prejudice and stigma in general [32], and more specifically in relation to mental illness, we argue for the need for further research exploring this correlation in a larger, more representative sample.

While a recent study has evaluated demographic factors as predictors of MHL in Lebanon [33], to our knowledge, this study is the first to evaluate digital health literacy, empathy, and mentalizing as predictors of MHL in Lebanon. It is also globally the first to hint that mentalizing could constitute a predictor of MHL, making mentalizing a new variable of interest. Moreover, it is the first to examine the positive impact of digital health literacy during the COVID-19 pandemic, an era that has considerably shifted health care practices from face-to-face to web-based. However, it is important to interpret the results in light of some limitations. First of all, since data collection took place in the midst of the COVID-19 pandemic, the sample size was small. Indeed, not many students participated in the study, possibly due to a lack of motivation and a multitude of web-based demands. Second, since the survey was disseminated on the web, only students comfortable with technology took part in the study. This may have biased our results, especially since we evaluate digital health literacy as a predictor of MHL. Indeed, Tohme et al [9] have shown that those who are familiar with social media platforms are more likely to seek help on the web through digital health channels. Finally, the MHLS-HS [3] is new, and its psychometric properties are not fully evaluated. For that, the results of this study would need to be replicated on a larger sample after having validated the scale.

In summary, our findings provide initial support for arguing the role of digital health literacy in fostering and promoting MHL. These findings should be replicated using different measures of digital literacy and MHL in a larger, more representative sample. If findings were to be supported, they could impact recommendations at the institutional, social, and personal levels. At the institutional level, it could hint that digital health literacy practices will become part of university curricula. As for the social level, it could give policymakers support to raise awareness about the importance of digital health and to offer digital health trainings to the general population, including people from different backgrounds, age groups, and socioeconomic status. Finally, at the personal level, health care providers could make a case for the use of digital health, such as telepsychotherapy, mobile health, and telehealth. Since this was a pilot study, in order to gain further insight and be able to generalize our results, it is recommended to replicate this study while collecting data from a larger sample, a sample that is not limited to students or people who have access to and are familiar with technology. Finally, given the significant correlations between health literacy and mental health outcomes [15], as well as our results highlighting the predictive role of some digital health literacy subscales on MHL, future research should evaluate whether digital health literacy can contribute to better mental health outcomes, an association that could be mediated by MHL.

## Abbreviations

BES: Basic Empathy Scale
eHLQ: eHealth Literacy Questionnaire
MHL: mental health literacy
MHLS-HS: Mental Health Literacy Scale for Healthcare Students
RFQ-8: Reflective Functioning Questionnaire-8
RFQc: Reflective Functioning Questionnaire-8–certainty about mental states
RFQu: Reflective Functioning Questionnaire-8–uncertainty about mental states

## Data Availability

The data sets generated during and/or analyzed during this study are available from the corresponding author upon reasonable request.
